# ERK5 Signalling and Resistance to ERK1/2 Pathway Therapeutics: The Path Less Travelled?

**DOI:** 10.3389/fcell.2022.839997

**Published:** 2022-07-12

**Authors:** Simon J. Cook, Pamela A. Lochhead

**Affiliations:** ^1^ Signalling Programme, The Babraham Institute, Babraham Research Campus, Cambridge, United Kingdom; ^2^ Mechanistic and Structural Biology, Discovery Sciences, R&D, AstraZeneca, Cambridge, United Kingdom

**Keywords:** ERK1/2, BRAF, MEK1/2, ERK5 MAP kinase, drug resisitance, PROTAC (proteolysis-targeting chimeric molecule), kinase inhibitors, oligonucleotide therapy

## Abstract

The RAS-regulated RAF-MEK1/2-ERK1/2 signalling pathway is frequently de-regulated in human cancer. Melanoma in particular exhibits a high incidence of activating BRAF^V600E/K^ and NRAS^Q61L/K^ mutations and such cells are addicted to the activity of these mutant oncoproteins. As a result three different BRAF inhibitors (BRAFi) have now been approved for BRAFV600E/K- mutant melanoma and have transformed the treatment of this disease. Despite this, clinical responses are typically transient as tumour cells develop resistance. These resistance mechanisms frequently involve reinstatement of ERK1/2 signalling and BRAFi are now deployed in combination with one of three approved MEK1/2 inhibitors (MEKi) to provide more durable, but still transient, clinical responses. Furthermore, inhibitors to ERK1/2 (ERK1/2i) have also been developed to counteract ERK1/2 signalling. However, recent studies have suggested that BRAFi/MEKi and ERK1/2i resistance can arise through activation of a parallel signalling pathway leading to activation of ERK5, an unusual protein kinase that contains both a kinase domain and a transcriptional transactivation domain. Here we review the evidence supporting ERK5 as a mediator of BRAFi/MEKi and ERK1/2i resistance. We also review the challenges in targeting ERK5 signalling with small molecules, including paradoxical activation of the transcriptional transactivation domain, and discuss new therapeutic modalities that could be employed to target ERK5.

## Introduction

**FIGURE 1:**
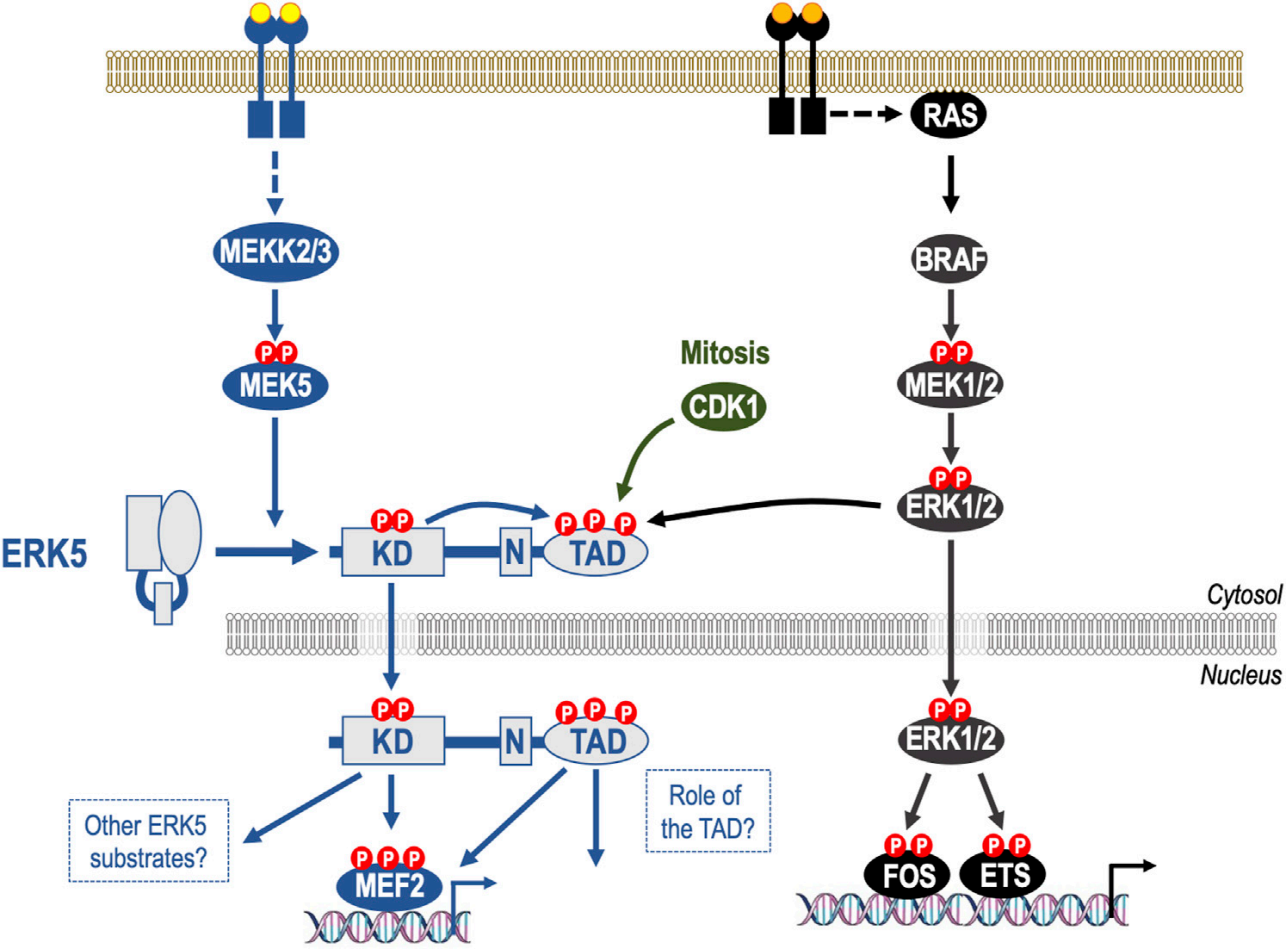
Mechanism of activation of ERK5 and its relationship to the canonical ERK1/pathway. The ERK5 signalling pathway is a three-tiered mitogen-activated protein kinase (MAPK) signalling cascade comprising the kinases MEKK2 and MEKK3 that phosphorylate and activate dual specificity kinase MEK5, which in turn phosphorylates the activation-loop T-E-Y motif in the ERK5 kinase domain, thereby activating it. Unlike ERK1/2, ERK5 has a large C-terminal extension that contains a nuclear localization signal (N) and a transcriptional transactivation domain (TAD). Upon kinase domain activation, ERK5 auto-phosphorylates multiple residues within its C-terminus, promoting nuclear localization of ERK5. This drives gene expression by direct phosphorylation of MEF2 transcription factors and by activation of the ERK5 TAD. Whilst MEK5 and MEK1/2 exhibit high sequence similarity MEK5 does not activate ERK1/2 and MEK1/2 do not activate ERK5; indeed, any effect of RAF-MEK1/2-ERK1/2 signalling on ERK5 activity is indirect and represents feed forward signalling or pathway cross talk. Indeed, some phosphorylation sites within the ERK5 C-terminus can also be phosphorylated by ERK1/2 and by CDK1 in mitosis. The C-terminal TAD can therefore integrate signals from both the ERK5 kinase domain and non-ERK5 kinases, including ERK1/2, to direct nuclear entry of ERK5.

The ERK1/2 signalling pathway consists of a three-tier hierarchical cascade of protein kinases in which RAF (ARAF, BRAF or CRAF) phosphorylates and activates the dual-specificity protein kinases MEK1 and MEK2, which in turn phosphorylate and activate ERK1 and ERK2 (ERK1/2) (Plotnikov et al., 2011). The RAS GTPases (HRAS, NRAS, and KRAS) play a key role in activating the pathway; activated, GTP-bound RAS recruits RAF proteins to the plasma membrane where they undergo phosphorylation-dependent activation (Lavoie and Therrien, 2015) (Figure 1). Once activated, ERK1/2 phosphorylates a variety of substrates to promote cell proliferation, cell survival and cell motility. These substrates include ETS and AP-1 transcription factors which drive expression of the D-type cyclins, thereby promoting progression through the G1 phase of the cell cycle (Meloche and Pouysségur, 2007). ERK1/2 signalling can also regulate members of the BCL-2 family of apoptotic regulators to promote cell survival (Cook et al., 2017; Sale et al., 2019).

**FIGURE 2:**
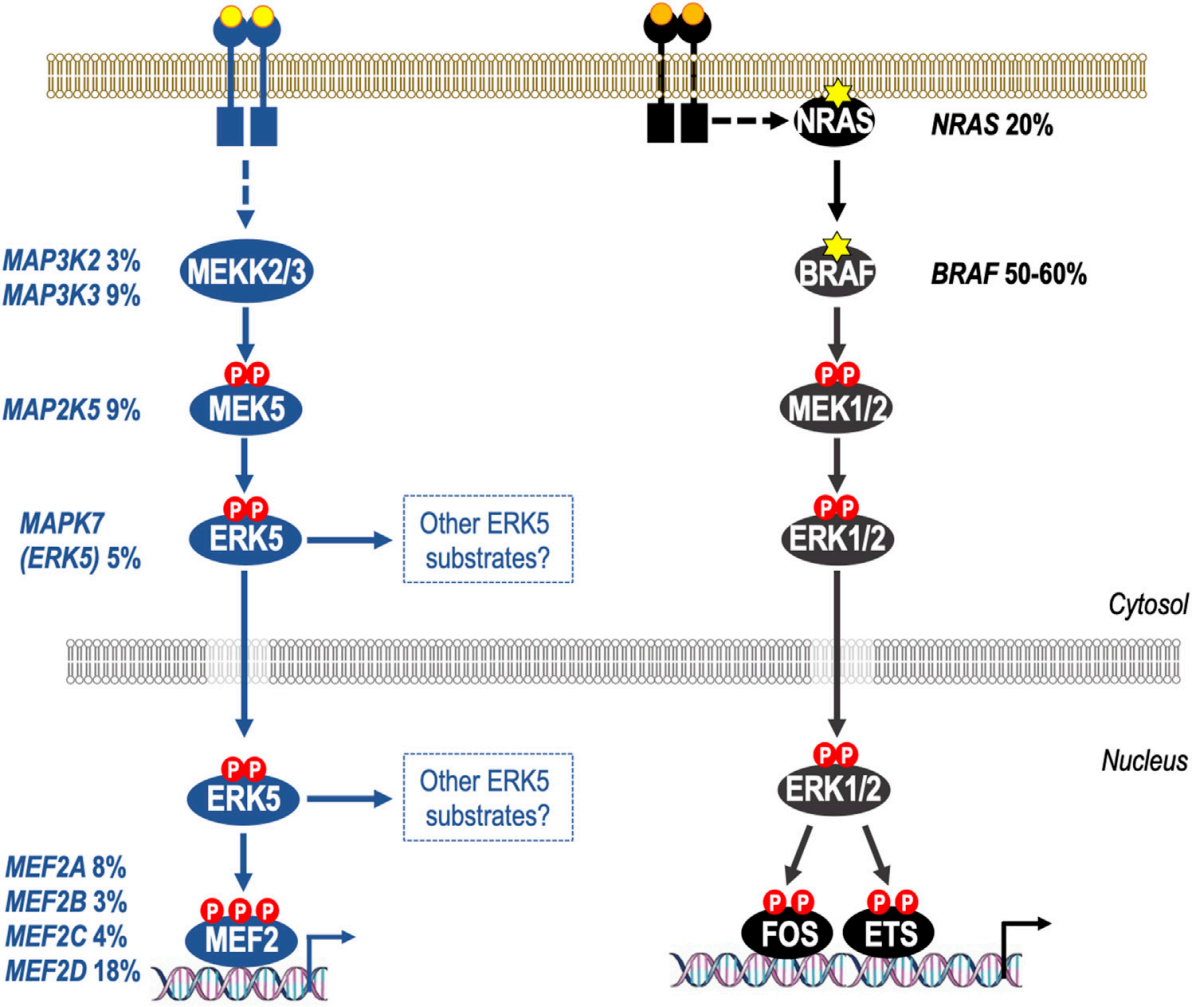
Activation of the ERK5 pathway through gene amplification and/or mRNA over-expression in melanoma. Both the ERK1/2 and ERK5 pathways are de-regulated in melanoma, but by distinct mechanisms. It is well known that the canonical ERK1/2 pathway is activated in melanoma through mutations in NRAS (20%) or BRAF (50%–60%). In the case of BRAF the vast majority of mutations are BRAF^V600E/K^ which cause constitutive activation of BRAF-MEK1/2-ERK1/2 signalling; melanoma cells harbouring BRAF^V600E/K^ are addicted to ERK1/2 signalling and BRAFi and MEK1/2i are now approved for treatment of BRAF^V600E/K^-mutant melanoma. More recently it has become apparent that the ERK5 pathway is also deregulated at high frequency in melanoma, with 47% of primary and metastatic melanomas exhibiting gene amplification and/or increases mRNA for ERK5 pathway components including MEKK2/3, MEK5, ERK5, and all four MEF2 transcription factors (MEF2A-D). Furthermore, shRNA against ERK5 reduces melanoma cell proliferation *in vitro* and melanoma growth in xenograft models suggesting that ERK5 therapies could have benefit in melanoma carrying either wild type BRAF or BRAF^V600E^ mutations.

**FIGURE 3:**
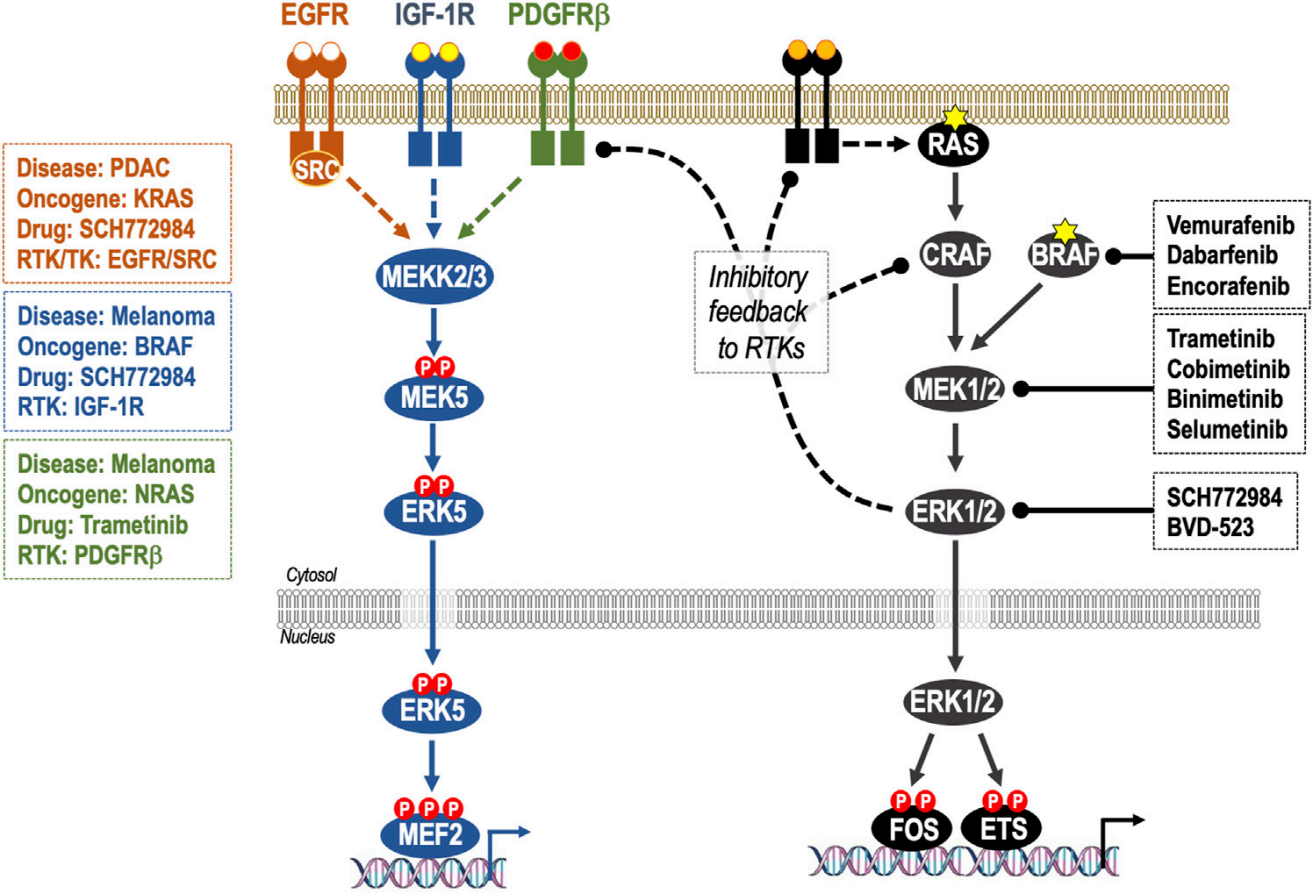
Adaptive resistance to ERK1/2 pathway inhibition through feedback relief, de-repression of RTKs and activation of ERK5. Activation of ERK1/2 cascade is controlled by homeostatic negative feedback loops operating at various levels including ERK1/2-dependent phosphorylation and inhibition of RAF and RTKs. When ERK1/2 signalling is inhibited by RAFi, MEK1/2i or ERK1/2i these inhibitory feedback loops are lost, resulting in reactivation of the pathway; this is manifest as rapid pathway “rebound” after initial pathway inhibition. Many of these same RTKs are also able to activate the ERK5 pathway, consequently loss of ERK1/2 activity can result in activation of the ERK5 pathway. This is seen in: KRAS-mutant PDAC, where inhibition of ERK1/2 results in activation of EGFR and SRC-dependent activation of ERK5; BRAF-mutant melanoma where inhibition of ERK1/2 results in activation of IGF-1R and thence ERK5 and in NRAS-mutant melanoma where inhibition of MEK1/2 results in activation of PDGFRβ and thence ERK5. In such cases, co-administration of ERK1/2 pathway therapeutics and ERK5i may be indicated.

ERK1/2 signalling is frequently de-regulated in human cancer due to mutations in receptor tyrosine kinase (RTKs), RAS (especially KRAS), and BRAF (Figure 2). As a result, the ERK1/2 pathway has attracted much interest in the search for new cancer therapeutics (Holderfield et al., 2014; Caunt et al., 2015) (Figure 3). Perhaps the best example has been the rapid development of BRAF inhibitors (BRAFi, such as vemurafenib and dabrafenib) for the treatment of melanoma (Bollag et al., 2010). BRAF is mutated in up to 60% of melanomas and most of these are activating BRAF^V600E^ or BRAF^V600K^ mutations. Melanoma cells harbouring BRAF^V600E/K^ are addicted to BRAF activity, explaining the striking clinical response to BRAFi (Bollag et al., 2010). They are in turn addicted to MEK1/2-ERK1/2 signalling, and this underpinned the approval MEK inhibitors (MEKi, such as trametinib and cobimetinib). Indeed, the combination of BRAFi + MEKi is now approved for treatment of melanoma with BRAF^V600E/K^. Despite this, clinical responses tend to be temporary, with disease relapse occurring due to the emergence of tumour cells with acquired resistance. The majority of mechanisms of acquired resistance to BRAFi and MEKi (alone or in combination) involve reinstating ERK1/2 signalling (Little et al., 2013; Holderfield et al., 2014; Caunt et al., 2015); these include BRAF amplification, the emergence of BRAF splice variants, a switch from BRAF to other RAF proteins or other MEK activators, the emergence of activating MEK1/2 mutations that circumvent the requirement for BRAF or MEK1/2 mutations that reduce or prevent MEKi binding. These mechanisms all support continued ERK1/2 signalling in the presence of BRAFi and/or MEKi, underlining the extent to which melanoma is addicted to this pathway. This has in turn driven the development of a range of highly selective ERK1/2 inhibitors (ERK1/2i), some of which are now undergoing clinical evaluation (Kidger et al., 2018). In addition to acquired resistance, innate resistance to ERK1/2 pathway inhibition is mediated by rapid pathway adaptation resulting from collapse of feedback loops. ERK1/2 regulates its own activity by negative feedback mechanisms, including direct inhibitory phosphorylation of upstream components such as MEK1/2, RAF, SOS, and RTKs (Lake et al., 2016). Consequently, whilst inhibition of ERK1/2 blocks downstream signalling, it also blocks this feedback inhibition, leading to rapid pathway reactivation that limits therapeutic effect.

More recently, studies have identified activation of the parallel ERK5 signalling pathway as a driver of BRAFi/MEKi (Song et al., 2017) and ERK1/2i resistance (Benito-Jardón et al., 2019) in melanoma and also as a driver of adaptive resistance to ERK1/2 pathway inhibition in pancreatic ductal adenocarcinoma (PDAC) (Vaseva et al., 2018) (Figure 3). Here we critically review the evidence supporting ERK5 as a mediator of BRAFi/MEKi and ERK1/2i resistance, both in BRAF-mutant melanoma and in RAS-driven tumours. We also review the challenges in targeting ERK5 signalling with small molecules, including the off-target effects of early ERK5 inhibitors (ERK5i) and the paradoxical activation of the transcriptional transactivation domain by ERK5i. Finally, we consider new therapeutic modalities that could be employed to target ERK5.

## The ERK5 Pathway as a Drug Target in Cancer

Like ERK1/2, ERK5 is the effector kinase of a three-tiered mitogen-activated protein kinase (MAPK) signalling cascade comprising the kinases MEKK2 and MEKK3 that phosphorylate and activate dual specificity kinase MEK5, which in turn phosphorylates the activation-loop T-E-Y motif in the ERK5 kinase domain, thereby activating it (Nishimoto and Nishida, 2006) (Figure 1). Whilst, MEK5 and MEK1/2 exhibit high sequence similarity, it is increasingly clear that these pathways are parallel, with few if any shared components. For example, MEK5 does not activate ERK1/2 and MEK1/2 do not activate ERK5. Furthermore, whilst early studies suggested that CRAF might directly activate ERK5 signalling (English et al., 1999), it now seems likely that any effect of RAF on ERK5 signalling is indirect and represents feed forward signalling or pathway cross talk (Lochhead et al., 2016) (see below). Consistent with this, whilst the kinase domains of ERK1/2 and ERK5 exhibit high sequence identity, they tend to have distinct substrates. For example, ERK1/2 phosphorylates members of the FOS family of transcription factors such as c-FOS and FRA1, whereas the role of ERK5 in c-FOS and FRA1 regulation remains unclear. ERK5 was originally proposed to phosphorylate and stabilise c-FOS and FRA1 and the C-terminal half of ERK5 was suggested to be necessary for maximal transactivation activity of c-FOS and FRA1 (Terasawa et al., 2003). However, a subsequent study showed that ERK5 activation was neither necessary nor sufficient for growth factor-stimulated expression or activation of c-FOS or FRA1 (Gilley et al., 2009). Whilst other ERK1/2 substrates have also been proposed as ERK5 substrates (Sap1a, c-MYC, RSK, and SGK) the best validated substrates of the ERK5 kinase domain remain the MEF2 family of transcription factors, which are not targeted by ERK1/2. Finally, the common phenotypes of MEK5 (Wang et al., 2005) or ERK5 (Regan et al., 2002) knockout bear no relation to the phenotypes of ERK1/2 or MEK1/2 knockout (Pagès and Pouysségur, 2004; Aouadi et al., 2006).

The unique and defining feature of the ERK5 cascade is ERK5 itself. Unlike ERK1/2, ERK5 has a large C-terminal extension that contains a nuclear localization signal and a transcriptional transactivation domain (TAD) (Lee et al., 1995; Zhou et al., 1995). Upon kinase domain activation, ERK5 auto-phosphorylates multiple residues within its C-terminus (Lochhead et al., 2012). Two of these sites, S753 and T732, have been characterised and phospho-specific antibodies have been raised against them (DÍaz-RodrÍguez and Pandiella, 2010). Both of these sites can also be phosphorylated by CDK1 (DÍaz-RodrÍguez and Pandiella, 2010; Iñesta-Vaquera et al., 2010; Gilley et al., 2012), whilst ERK1/2 can phosphorylate T732 (Honda et al., 2015). Phosphorylation of the C-terminal sites drives nuclear localization of ERK5 and activation of the TAD (Buschbeck and Ullrich, 2005; Morimoto et al., 2007). The presence of a C-terminal TAD that can integrate signals from both the ERK5 kinase domain and non-ERK5 kinases, including ERK1/2, to direct nuclear entry of ERK5 may have important implications for ERK5 signalling in tumours cells with deregulated ERK1/2 signalling and their response to therapeutics (Figure 1).

Early cellular studies indicated that like the RAF-MEK1/2-ERK1/2, the MEK5-ERK5 pathway was important for proliferation, survival and motility, contributing to specific cancer hallmarks (Lochhead et al., 2012). More recently other reviews have assessed the viability of ERK5 as a drug target in cancer (Simões et al., 2016; Tubita et al., 2020). There is strong evidence that ERK5 drives cancer cell motility but the role of ERK5 in driving cell proliferation is cancer cell type-dependent and the role of ERK5 in cell survival is less well understood (Lochhead et al., 2012; Hoang et al., 2017; Stecca and Rovida, 2019).

The clinical evidence seems clear that increased expression of ERK5 in tumours results in poor prognosis for patients. A decrease in overall and disease-free survival for patients with elevated ERK5 levels has been seen in prostate cancer (Mehta et al., 2003), breast cancer (Ortiz-Ruiz et al., 2014; Miranda et al., 2015; Xu et al., 2021), lung cancer (Jiang et al., 2020; Sánchez-Fdez et al., 2021), colorectal cancer (Simões et al., 2015; Pereira et al., 2016), glioma (Carmell et al., 2021), osteosarcoma (Tesser-Gamba et al., 2012) and melanoma (Tusa et al., 2018). A recent study by Monti et al. (2022) analysed The Cancer Genome Atlas dataset and found that ERK5 expression was variable across tumour type, but patients with high ERK5 expression were associated with worse overall survival time. However, functional studies to define how ERK5 drives this poor prognosis have been plagued by off-target effects of early ERK5 kinase inhibitors (ERK5i), most notably against bromo-domain containing proteins (Lin et al., 2016), and confounding paradoxical activation of ERK5 signalling. These matters are described in detail later in this review but for these reasons it is increasingly important that studies that assess the therapeutic potential of targeting ERK5 with ERK5i are confirmed using MEK5i and/or independent genetic interventions such as RNA interference or gene knock-out. Studies that have deployed knockdown or knockout of ERK5 (or MEK5) by siRNA have shown therapeutic potential for ERK5 in prostate cancer (Clapé et al., 2009), bladder cancer (Noguchi et al., 2011), breast cancer (Ortiz-Ruiz et al., 2014), KRAS-dependent pancreatic ductal adenocarcinoma (PDAC) (Vaseva et al., 2018), and in melanoma (Hayashi et al., 2005; Giurisato et al., 2018; Tusa et al., 2018; Giurisato et al., 2020). Here we focus on studies in melanoma and PDAC.

## Evidence for ERK5 as an Oncogenic Driver Pathway in Melanoma

The role of the ERK5 pathway in melanoma tumour cells is starting to be revealed, and it is hoped that this will allow an understanding of how ERK5-targeted therapies may be used in the clinic. Indeed, there is promise that targeting ERK5 will be an effective strategy both in altering the tumour microenvironment and by directly preventing tumour cell growth. Gene targeting strategies to knock-out ERK5 in tumour-associated macrophages impedes the growth of melanoma (and lung carcinoma) in mouse models (Giurisato et al., 2018; Giurisato et al., 2020), and whole mouse conditional ERK5 knockout reduces tumour neo-vascularization of a melanoma xenograft (Hayashi et al., 2005). In tumour cells efficient ERK5 gene silencing using RNAi techniques prevents cell proliferation of melanoma cell lines (Tusa et al., 2018) and xenograft tumour growth (Song et al., 2017; Tusa et al., 2018; Benito-Jardón et al., 2019; Adam et al., 2020).

Whereas, for the ERK1/2 pathway, specific somatic mutations in BRAF or NRAS (such as BRAF^V600E^ or NRAS^Q61K/L^) cause ERK1/2 pathway activation and drive tumour cell proliferation, survival and motility, somatic mutations in ERK5 signalling pathway components are rare and none have been detected with high frequency. Instead it appears that upregulation of mRNA levels and/or gene amplification of ERK5 pathway components is what drives tumour cell dependency. For example, Tusa et al. (2018) analysed data from melanoma patients and found that 223 out of 479 (47%) primary and metastatic melanomas had gene copy number and mRNA alterations in ERK5 pathway components at all levels in the pathway including MEKK2/3, MEK5, ERK5 or MEF2A-D. This compares with 50%–60% patients with mutations in BRAF or 20%–25% in NRAS (Figure 2). Melanoma cells with BRAF^V600E^ or wild type BRAF showed a reduction in tumour cell proliferation *in vitro* and tumour growth in xenograft models when using shRNA against ERK5 (Tusa et al., 2018), suggesting that ERK5 therapies could have benefit in tumours carrying either wild type BRAF or BRAF^V600E^ mutations. An analysis of signalling showed that BRAF^V600E^-mutant cells exhibited enhanced phosphorylation of the ERK5 activation-loop T-E-Y motif, as well as residues in the C-terminal transactivation domain (S753 and T732) (the role of which is discussed above). The BRAF^V600E^-driven increase in ERK5 activation-loop T-E-Y phosphorylation was weak compared to the level of phosphorylation driven by MEK5D (the constitutively active form of the ERK5 activating kinase MEK5), as seen in other studies (Lochhead et al., 2016), but the C-terminal sites were induced to a similar level as that with MEK5D. All these sites were sensitive to ERK1/2i, with SCH772984 causing a reduction in phosphorylation, and a further reduction was seen when SCH772984 was co-administered with the CDK1 inhibitor RO-3306, demonstrating that ERK1/2 and CDK1 are required for their phosphorylation. Consistent with phosphorylation of the transactivation domain, BRAF^V600E^ also increased nuclear and chromatin bound ERK5 and an increase in transcriptional activity (Tusa et al., 2018). The low level of ERK5 activation-loop T-E-Y phosphorylation induced by BRAF^V600E^ was only partially responsive to SCH772984 and RO-3306 alone or in combination. Therefore, quite how the serine/threonine kinases ERK1/2 and CDK1 are contributing to T281/Y220 phosphorylation remains unclear; is this directly on T218 only; or if indirectly, how do they stimulate MEK5? Or are other kinases involved?

## Evidence for ERK5 as a Mediator of Innate and Acquired Resistance to BRAF-MEK1/2-ERK1/2 Pathway Inhibitors

The combination of BRAFi and MEKi is approved for melanoma, but acquired resistance to this combination therapy is common (Chapman et al., 2014; Holderfield et al., 2014; Caunt et al., 2015). Using SILAC labelling of cellular proteins and siRNA gene silencing, ERK5 was identified as a mediator of BRAFi (vemurafenib) and MEKi (trametinib) resistance (Song et al., 2017). Interestingly, combined treatment with BRAFi and MEKi caused an increase in ERK5 phosphorylation as quickly as 1 h after treatment and this persisted in resistant cells and xenograft models. ERK5 activation (using MEK5D) desensitized treatment-naïve cells to the BRAFi + MEKi combination, while shRNA-mediated ERK5 silencing sensitized these cells. Furthermore, shRNA-mediated silencing of ERK5 greatly reduced the outgrowth of BRAFi/MEKi resistant colonies (Song et al., 2017). These studies provide strong evidence for ERK5 as a mediator of BRAFi + MEKi resistance in melanoma.

A further study found that the ERK5 pathway also mediated acquired resistance to the experimental ERK1/2i, SCH772984 (Benito-Jardón et al., 2019). In ERKi-resistant melanoma cells ERK1/2 signalling was downregulated with decreased phosphorylation of MEK1/2, ERK1/2, and p90-RSK. In contrast, ERK5 phosphorylation was increased (and in some cell lines ERK5 protein levels were also increased), together with an increase in the mRNA levels of the ERK5 target genes c-MYC and c-JUN. Consistent with Tusa et al. (2018), BRAF^V600E^-mutant melanoma cells were sensitive to ERK5 pathway inhibition using the MEK5 inhibitor, BIX02189 (Benito-Jardón et al., 2019). Furthermore, ERK1/2i-resistant cells were more sensitive to ERK5 pathway inhibition than treatment-naïve cells. These results were confirmed using shRNA to ERK5 or expression of MEK5A—a dominant negative form of the ERK5-activating kinase MEK5 in which the activation-loop phosphorylation sites, required to be phosphorylated for MEK5 activity, are mutated to alanine to prevent phosphorylation, creating a non-activatable form of MEK5. These authors also established that insulin-like growth factor receptor 1 (IGF-1R) was upregulated (by stabilization) and ERK1/2i-resistant melanoma cells were dependent on IGF1-R activity (using the IGF1-R inhibitor, linsitinib) for cell proliferation *in vitro*, in spheroids and in xenograft models. Treatment-naïve melanoma cells were sensitive to IGF1-R inhibition but ERK1/2i-resistant cells much more so, like their increased sensitivity to ERK5 pathway inhibition; indeed, ERK5 activation in resistant cells was IGF1-R-dependent (Figure 3). This provides more evidence that ERK5 is a key player in mediating resistance to ERK1/2 pathway inhibition in melanoma. In the study by Song et al. (2017) using BRAFi and MEKi, an increase in IGF1-R phospho-peptides were not detected in their SILAC experiment. This suggests that ERK5 can be influenced by different pathways and demonstrates that ERK5 therapeutics may have benefit in a range of resistance settings in melanoma.

A really interesting observation made in this study (Benito-Jardón et al., 2019) is the difference in mechanism of resistance depending on whether ERK1/2T-E-Y phosphorylation is inhibited. BRAFi (vemurafenib)-resistant BRAF^V600E^ cells adapted by reinstating the same level of phophorylated ERK1/2 as was seen in treatment-naïve parental cells. This has also been observed in colorectal cancer cells with BRAF^V600E^ that were rendered resistant to the MEKi, selumetinib, where ERK1/2 signalling must be maintained within a “sweet spot” to maintain proliferation (Little et al., 2011; Sale et al., 2019). These BRAFi-resistant melanoma cells did not exhibit an increase in phosphorylated ERK5. In contrast, cells rendered resistant to BRAFi (vemurafenib) plus MEKi (trametinib) or BRAFi (vemurafenib) plus ERK1/2i (SCH772984) exhibited decreased phosphorylated ERK1/2 but increased phosphorylated ERK5 and were sensitive to the MEK5 inhibitor, BIX02189 (Benito-Jardón et al., 2019). One thing that MEKi and the ERK1/2i, SCH772984 have in common is that they both prevent the T-E-Y phosphorylation of ERK1/2. MEKi’s inhibit MEK1/2, the ERK1/2 activation-loop kinase, thereby preventing phosphorylation of ERK1/2 at T-E-Y. SCH772984 is a dual-mechanism ERK1/2i that both inhibits ERK1/2 catalysis and prevents T-E-Y activation-loop phosphorylation by MEK1/2 (Deng et al., 2014). Therefore it may be the lack of phosphorylated ERK1/2 and/or the inactive conformation of ERK1/2 that triggers activation of the ERK5 pathway. There is a growing appreciation of the role of kinase conformation, other than simple catalytic activity, in mediating cellular signalling events (Byrne et al., 2017; Kung and Jura, 2019).

NRAS^Q61L/K^-driven melanomas show a high rate of innate resistance to MEKi (Dummer et al., 2017). A recent study by Adam et al. (2020) investigated the involvement of the ERK5 pathway in mediating this resistance. They found that after 2 days treatment with MEKi (trametinib) phosphorylated ERK5 (the activation-loop T-E-Y motif) was increased. This was also seen with three other MEKi’s, binimetinib, selumetinib, and cobimetinib. Unlike BRAF^V600E^-driven melanoma cells, shRNA-mediated silencing of ERK5 in NRAS^Q61L/K^ driven melanoma had no effect on its own; however, a combination of MEKi and shERK5 decreased cell viability and this result was confirmed with the MEK5 inhibitor, BIX02188. Unlike melanoma cells harbouring BRAF^V600E^ that upregulated IGF1-R in response to ERK1/2i (Benito-Jardón et al., 2019), melanoma cells harbouring NRAS^Q61L/K^ increased PDGRβ, and the increase ERK5 phosphorylation was sensitive to the PDGFRi’s, crenolanib, and CP673451 (Figure 3).

Taken together these findings provide strong evidence that ERK5 pathway therapeutics may have use in the treatment of melanoma in combination with BRAF-MEK1/2-ERK1/2 pathway inhibitors to overcome intrinsic resistance and hinder or delay some modes of acquired resistance. They also suggest that ERK5 activation arising from ERK1/2 pathway inhibition may involve engagement of RTKs including IGF-1R or PDGFR, which are both druggable targets. The challenge now is to develop small molecules that can effectively prevent ERK5 pathway signalling.

## Evidence for Adaptive Resistance to ERK1/2 Inhibition in PDAC by ERK5

It has long been known that the transcription factor MYC is required for KRAS-driven PDAC (Land et al., 1983; Soucek et al., 2013; Saborowski et al., 2014; Walz et al., 2014). Recently Vaseva et al.(2018) found that the ERK5 pathway regulates MYC protein levels in a feed-forward mechanism when ERK1/2 is inhibited. This is dependent on EGFR and SRC (Figure 3). They started by showing that acute suppression of KRAS by siRNA caused MYC loss and this was mediated by loss of MEK1/2-ERK1/2 signalling, assessed using the MEKi and ERKi, selumetinib, and SCH772984, respectively. However, knockdown of the MYC E3 ligase FBXW7 prevented the transient loss of MYC by ERK1/2 and MEK1/2 inhibition, but not that mediated by KRAS knockdown suggesting that there were additional KRAS-driven pathways that regulate MYC protein levels. To identify these pathways they took 3 approaches. First, they conducted a kinome-wide chemical proteomics screen using multiplex kinase inhibitor beads and mass spectrometry (MIB/MS) following KRAS knock down by siRNA; up regulation of MEK5 was identified in 3 PDAC cell lines. The second approach was a gain-of-function screen using the CANCER Toolkit (lentivirus expression vector-based library encoding 36 activated components representing 17 different cancer signalling pathways) (Martz et al., 2014) to screen for the stabilisation of a fluorescence-based sensor to monitor MYC stability. From this two active forms of MEK5 were identified; a myristoylated MEK5 (which tethers MEK5 to the plasma membrane) and the constitutively active form of MEK5, MEK5D. Finally, the Published Kinase Inhibitor Set (PKIS) and PKIS2 libraries of ATP-competitive protein kinase inhibitors (Elkins et al., 2016; Drewry et al., 2017) were screened for their ability to regulate MYC protein stability; this identified a molecule (UNC10225170) that can inhibit MEK5. The role of MEK5 and ERK5 was confirmed by over-expression of ERK5, which reduced polyubiquitination and increased MYC half-life and ERK5 siRNA or MEK5 inhibition by BIX02819, which caused proteasome-dependent loss of MYC.

So how does KRAS knockdown lead to MEK5-ERK5 activation? Inhibition of MEK1/2 or ERK1/2 with selumetinib or SCH772984 also lead to increased ERK5 phosphorylation. ERK1/2 regulates its own activity by negative feedback mechanisms acting at the level of MEK1/2, RAF, SOS, and RTKs to inhibit ERK1/2 activation; consequently, inhibition of ERK1/2 blocks this feedback inhibition leading to pathway reactivation (Lake et al., 2016). This is seen for growth factor receptor and tyrosine kinase signalling pathways that are also able to activate the MEK5-ERK5 pathway. Indeed, the ERK1/2i induced-activation of ERK5 phosphorylation was inhibited by EGFR or SRC inhibition with small molecules (poziotinib or erlotinib, and saracatinib, respectively), showing ERK5 activation resulted from activation of RTK signalling. This is similar to ERK5 activation by RTKs in melanoma when MEK1/2 or ERK1/2 signalling is inhibited; through IGF-1R in BRAF^V600E^ driven melanoma resistant to the ERKi SCH772984 (Benito-Jardón et al., 2019) or through PDGRβ in NRAS^Q61L/K^ driven melanoma resistant to the MEKi trametinib (Adam et al., 2020) (see above and Figure 3).

**FIGURE 4:**
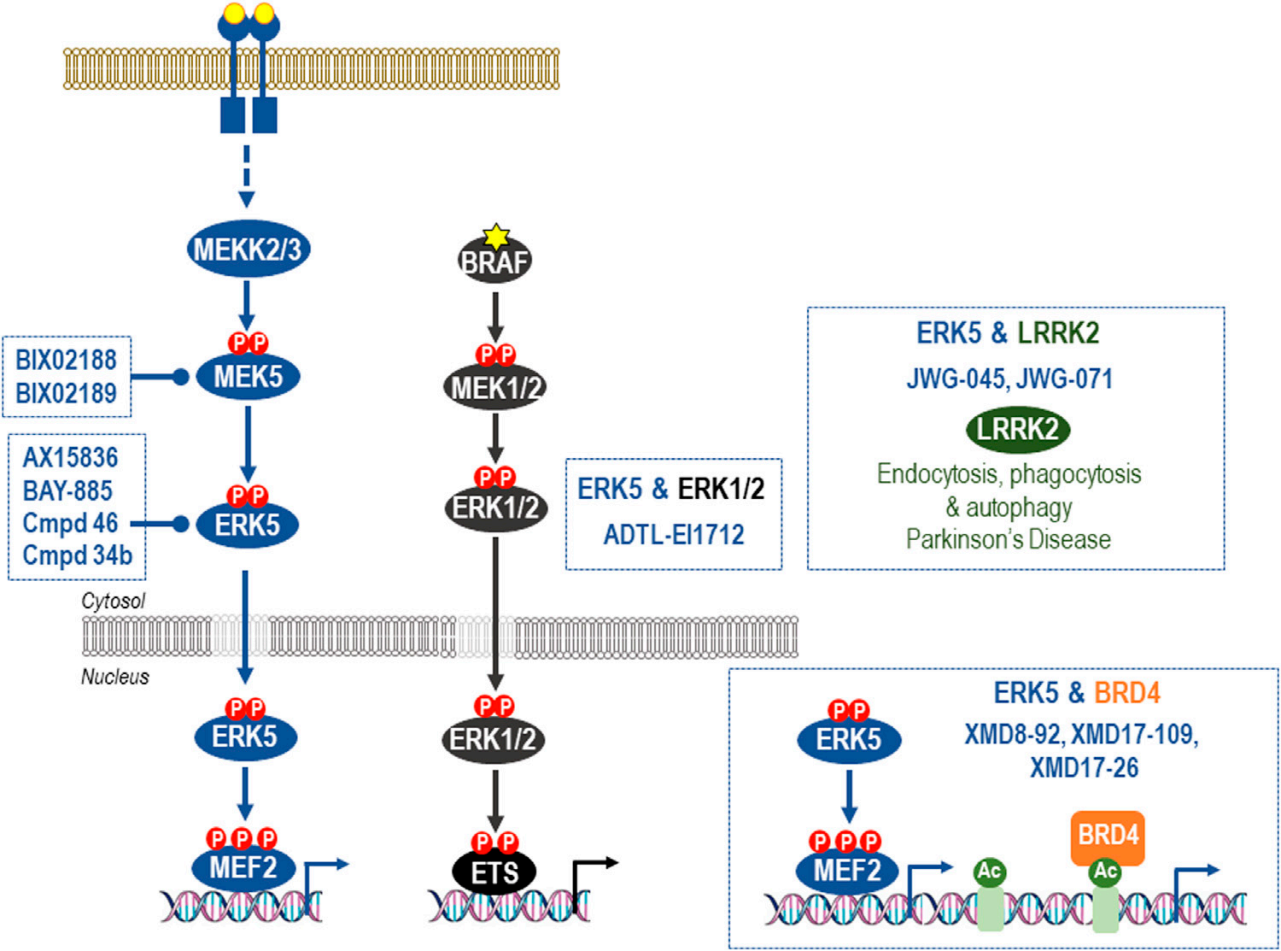
Off-target effects of common ERK5 inhibitors. The earliest ERK5 inhibitor, XMD8-92, has been used extensively to probe supposed roles of ERK5 kinase activity. However, XMD8-92 also inhibits binding of BRD4 to its target acetylated protein targets to modulate transcriptional processes. Indeed, XMD8-92 is equipotent for inhibition of ERK5 kinase activity and blockade of binding of BRD4 to ε-*N*-acetylated lysine-containing sequences. This “off-target” activity against a critical regulator of gene expression is shared by the closely related XMD17-109 and XMD-17-26 and likely accounts for the anti-inflammatory and anti-proliferative effects of these agents. For this reason they should no longer be employed in cellular or *in vivo* studies of ERK5 function and studies that have relied upon them should be revisited and viewed with caution. Second generation ERK5i that lack BRD4 activity have now been described and include the dual-ERK5/LRRK2 inhibitors, JWG-045 (XMD10-78), and JWG-071. ADTL-EI1712 inhibits ERK1, ERK2, and ERK5 and may find utility in the treatment of melanoma where resistance to BRAFi, MEKi or ERKi is sometimes driven activation of ERK5; however, it is not known if ADTL-EI1712 has BRD4 activity. AX15836, compound 46, and BAY-885 lack BRD4 binding activity and are currently the most potent and selective inhibitors of ERK5 kinase activity and the preferred options for selective inhibition of ERK5 kinase activity in cells and *in vivo*. Finally, ERK5 activation can also be prevented by the potent and selective MEK5 inhibitors BIX02188 and BIX02189.

Finally, the authors observed PDAC cell growth inhibition when ERK5 siRNA or MEK5i were combined with ERK1/2 inhibition (SCH772984), and this was mirrored by MYC loss (Vaseva et al., 2018). This study also employed XMD8-92 to demonstrate inhibition of growth of PDAC tumour xenografts in combination with ERK1/2i; given the dual-ERK5/BRD4 activity of XMD8-92 it will be critical to see these experiments repeated with a selective ERK5i that does not have BRD4 activity (for more detail see below and Figure 4) (Vaseva et al., 2018).

The study by Vaseva provides one example of how ERK5 may contribute to the maintenance of PDAC. However, in contrast to the growing body of evidence in melanoma and other cancers, the rationale for ERK5 inhibition in PDAC is, as yet, less well advanced. Given the high unmet clinical need in PDAC, further work is urgently required here.

## Challenges of Targeting ERK5

### Off-Target Effects of ERK5 Inhibitors

Much of the preclinical work to assess the therapeutic potential of ERK5i to date has employed the small molecule inhibitor XMD8-92. This was the first ERK5i to be described but was subsequently shown to have significant off-target effects on BRD4, a bromo-domain containing protein that recognises acetylated lysine residues and plays a role in regulating gene transcription (Lin et al., 2016). As a result, XMD8-92 is not a suitable tool to delineate the cellular role of ERK5 kinase activity. For example, a study implicating ERK5 in promoting cancer stem cell-like properties and tumour-sphere growth in colorectal cancer relied heavily on the use of XMD8-92 (Pereira et al., 2019) making it unclear if the study is reporting ERK5 dependency, BRD4 dependency or both. Much the same applies to the use of XMD8-92 to inhibit growth of PDAC tumour xenografts (Vaseva et al., 2018). Subsequent derivatives of XMD8-92 including XMD17-109 (compound26, ERK5-IN-1), and XMD17-26 (compound 25) also exhibit off-target activity against BRD proteins [reviewed in (Cook et al., 2020)]. For these reasons it is critical that studies that assess the therapeutic potential of targeting ERK5 employ more selective ERK5i and confirm results using MEK5i and/or independent genetic interventions such as RNA interference or gene knock out.

AX15836 was the first ERK5i to be identified that lacks BRD4 binding activity and was used to demonstrate that ERK5 kinase domain inhibition does not phenocopy genetic ablation of ERK5, leading to the suggestion that ERK5’s large C-terminus contributes to certain ERK5 cellular functions (Lin et al., 2016). Thus RNAi to ERK5 and XMD8-92 seem to function in different ways; RNAi removes the ERK5 protein, including the kinase domain and the C-terminal transcriptional transactivation domain, whilst XMD8-92 acts through the combined inhibition of the ERK5 kinase domain and BRD4, or just through inhibition of BRD4. For example, ERK5 and BRD4 converge on regulation of the KLF2 promoter: ERK5 is known to regulate KLF2 promoter activity through phosphorylation of MEF2 transcription factors; however, the KLF2 promoter is also sensitive to the BRD4 inhibitor, JQ1 (Lochhead et al., 2020). For these reasons all new ERK5i should be tested for activity against BRD4. In addition, XMD8-92, XMD17-109, and XMD17-26 should not be used to evaluate the cellular or *in vivo* role of ERK5 kinase activity, whilst studies that have relied alone or in large part on these drugs should be re-evaluated.

More recently a variety of second generation ERK5i that lack BRD4 activity have been described [reviewed in (Cook et al., 2020)]. These include the dual-ERK5/LRRK2 inhibitors, JWG-045 (XMD10-78), and JWG-071 (Wang et al., 2018), and the highly selective ERK5i, AX15836 (Lin et al., 2016), compound 46 (Myers et al., 2019), and BAY-885 (Myers et al., 2019). At present AX15836, compound 46, and BAY-885 are the preferred options for selective inhibition of ERK5 kinase activity. The selectivity of all these inhibitors is summarised in Figure 4.

### Paradoxical Activation of the Transactivation Domain

**FIGURE 5:**
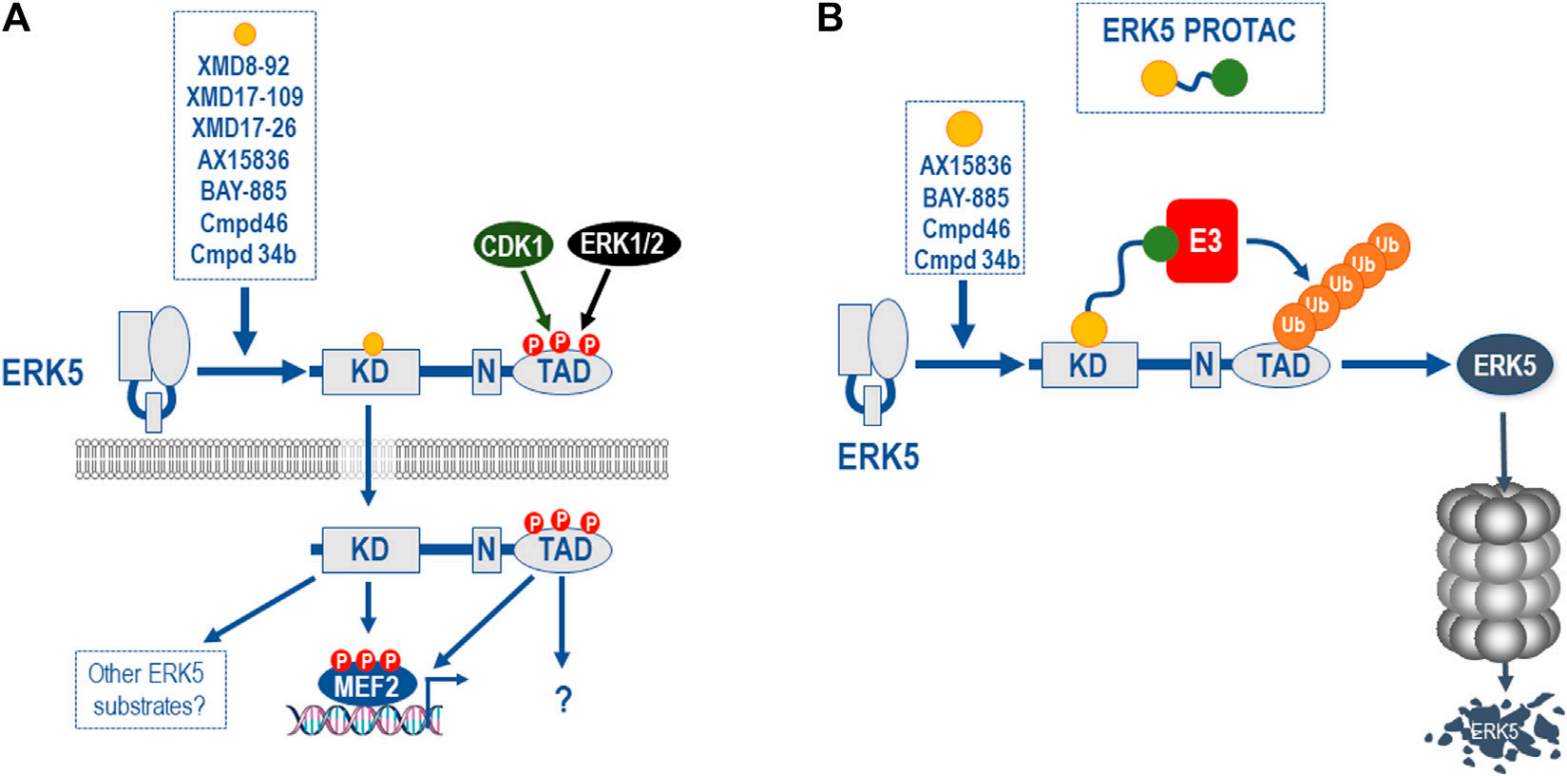
Paradoxical activation of the ERK5 C-terminal TAD function by ERK5i and ERK5 PROTACS as a potential solution. **(A)** All ERK5i tested to date, including XMD8-92, XMD17-109, XMD17-26, AX15836, compound 46, compound 34b, and BAY-885 (and represented by orange circle) have been shown to bind to the ERK5 kinase domain and cause a conformational change that leads to the exposure of the NLS, nuclear localisation, and paradoxical activation of the C-terminal TAD, albeit to varying degrees (Cook et al., 2020; Lochhead et al., 2020). This is seen with AX15836, compound 46, and BAY-885 as well as XMD8-92 so it is unrelated to BRD4 activity. The fact that this is seen at doses that inhibit ERK5 kinase activity indicates that this is a kinase-independent activity of ERK5 and is consistent with kinase independent biological effects of ERK5 (Lin et al., 2016). **(B)** Since ERK5 possesses both kinase-dependent and -independent functions and the latter are not targeted by conventional ERK5 kinase inhibitors, other approaches are required to fully inhibit the various biochemical functions of ERK5. One attractive approach is to a develop proteolysis targeting chimeras (PROTAC) in which a potent, selective ERK5 ligand (represented by orange circle) is linked to an E3 ubiquitin ligase recruiting ligand (represented by green circle), resulting in the poly-ubiquitylation and degradation of ERK5. This approach will ablate all ERK5 functions including catalytic activities, allosteric regulatory sites, scaffolding or protein-protein interaction sites and the TAD function. The availability of potent and selective ERK5i (AX15836, compound 46, compound 34b, and BAY-885) may allow the relatively rapid development and testing of an ERK5 PROTAC.

Even the second generation ERK5i face a further challenge in development. A range of ERK5 kinase domain inhibitors have recently been shown to bind to ERK5 and cause a conformational change that leads to the exposure of the NLS, nuclear localisation, and paradoxical activation of the C-terminal TAD (Lochhead et al., 2020). All ERK5i tested to date, including XMD8-92, XMD17-109 (compound 26, ERK5-IN-1), XMD17-26 (compound 25), AX15836, compound 46, BAY-885, and compound 34b, cause paradoxical activation of the ERK5 TAD, albeit to varying degrees (Cook et al., 2020; Lochhead et al., 2020; Miller et al., 2022) (Figure 5A). These inhibitors are type I (that is, they bind the active kinase conformation) ATP-competitive inhibitors, and it remains possible that other classes of ERK5i may not have the same effect.

ADTL-EI1712 is a new class of inhibitor that inhibits ERK1, ERK2, and ERK5 (Wang et al., 2020) (Figure 4). This inhibitor might find utility in the treatment of melanoma where resistance to BRAFi, MEKi or ERKi is sometimes driven by activation of ERK5 (see above). ADTL-EI1712 is also a type I, ATP-competitive inhibitor but it is not known if it has BRD4 activity or if it paradoxically activates the ERK5 TAD.

The discovery of paradoxical activation of the ERK5 TAD by ERK5 kinase inhibitors brings the function of the ERK5 C-terminus sharply into focus and raises fundamental questions. What biological effects of ERK5 KO reflect loss of kinase activity, loss of the C-terminal TAD function or both? What are the functions of naturally occurring ERK5 splice variants (Yan et al., 2001; McCaw et al., 2005; Monti et al., 2022) that seem to lack the C-term but are predicted (by the presence of key kinase domain motifs) to retain kinase activity. Will these variants support kinase-dependent MEF2 activation that is fully inhibited by ERK5i? What biological functions are controlled by the ERK5 C-terminal extension, including the TAD and are these all kinase-dependent? Another splice variant retains the NLS and TAD but lacks the N-terminus of the kinase domain, and therefore should lack kinase activity, raising the possibility that the NLS and TAD have kinase-independent functions. These questions are not only important for understanding the normal biological role of this unusual kinase-transcription factor hybrid but are also critical for understanding how best to inhibit its biological activity. Such strategies should more faithfully phenocopy genetic ablation of ERK5 and will also help to define whether ERK5 ablation rather than ERK5 kinase inhibition is a more desirable therapeutic avenue.

## Are Therapies That Modulate ERK5 Protein Abundance the Answer?

The protein kinase domain is amenable to small molecule inhibitors. The first protein kinase inhibitor to be approved for use in the clinic was Imatinib more than 20 years ago. Since then more than 70 protein kinase inhibitors have been approved for clinical use (Cohen et al., 2021). Therefore, it is not surprising that when seeking to target ERK5 in cancer (or other indications) there has been a lot of interest in developing small molecule inhibitors of the kinase domain, including our own collaborations with excellent drug discovery teams (Myers et al., 2019; Miller et al., 2022). However, kinase inhibitor programmes have experienced common setbacks including 1) innate or acquired resistance to the kinase inhibitor due to mutation of the intended target, mutation of other pathway components or pathway remodelling (as exemplified above in melanoma and PDAC) and 2) unintended activation of the target pathway, either by inhibition of negative feedback pathways or through inhibitor binding to the kinase resulting in paradoxical activation (the latter termed “inhibitor hijacking of kinase activation”). Unfortunately a range ERK5i are capable of inducing a paradoxical effect on the ERK5 TAD function (Cook et al., 2020; Lochhead et al., 2020). At present AX15836, compound 46, compound 34b, and BAY-885 are the most potent and selective inhibitors of ERK5 kinase activity but they are all able to drive paradoxical activation of the C-terminal TAD (Lochhead et al., 2020; Miller et al., 2022). Clearly a priority is to develop selective (non-BRD4 binding) ERK5i that act as “paradox breakers” by inhibiting kinase activity without causing paradoxical activation of the TAD. These will be useful in defining the biological roles of the ERK5 kinase domain and C-terminal extension and TAD. However, the wider issue is that ERK5 appears to exhibit both kinase-dependent and kinase-independent functions and the latter appears not to be targeted by conventional ERK5 kinase inhibitors. Clearly other approaches are required to effectively inhibit all the various biochemical functions of ERK5.

There is a growing appreciation that many enzymes, including protein kinases, also contain other functional domains that are critical to the sum of the protein’s function, including those involved in protein-protein interactions (PPIs) or directing a discrete sub-cellular localisation. In addition, some enzymes apparently lack classical enzymatic activity but retain other functional domains and harness co-factor binding to control them; for example, pseudokinases fail to catalyse a phosphotransfer reaction but use ATP binding to drive conformational changes that facilitate signal transmission by promoting PPIs. With this has come the recognition of the need for novel ways to inhibit these proteins, beyond traditional small molecule enzyme inhibitors. These “new modalities” include antibodies, peptides, oligonucleotides, hybrids, and molecular conjugates and can be used to inhibit protein-protein interactions, downregulate targets or stabilise them [reviewed in (Valeur et al., 2017)]. Here we consider two ways to downregulate the expression of ERK5, thereby removing the kinase and non-kinase functions of the protein. These are Proteolysis-Targeting Chimeras (PROTACs) and Oligonucleotide Therapies, both of which rely on cellular machinery, independent of the therapeutic target, to mediate their effects. These new therapies also face challenges of uptake into the cells and getting to the disease location within the body. Thus, careful consideration should be given to how they will target ERK5 in disease relevant cells.

### Proteolysis-Targeting Chimeras

The last few years have seen an explosion of interest in proteolysis targeting chimeras (PROTACs) as a strategy to drive the degradation of proteins rather than inhibiting certain functions (Chamberlain and Hamann, 2019; Bekes et al., 2022). PROTACs are hetero-bifunctional molecules consisting a ligand for an intracellular target protein-of-interest (POI) and an E3 ubiquitin ligase ligand, joined by a linker which brings the POI and E3 ligase together; this drives polyubiquitylation of the POI and its subsequent degradation by the 26S proteasome. PROTACs offer an alternative approach over classical small molecule inhibitors with some notable advantages. Since the POI is degraded, the PROTAC is recycled to target another copy of the POI. This catalytic mode of action is termed “event-driven pharmacology” and sets it aside from the classical one-to-one target-to-inhibitor interaction. Also, by driving destruction of the target POI, PROTACs should provide a more durable effect that will only be reversed by cellular de-ubiquitylase activity or resynthesis of the target POI. Perhaps more importantly, in the context of ERK5, a small molecule inhibitor such as a protein kinase inhibitor typically only targets one function of a protein, whereas the degradation of the protein ablates all functions including catalytic activities, allosteric regulatory sites and scaffolding or protein-protein interaction sites. This may lead to a more pronounced phenotype than targeting just one domain or function; whether this results in too severe a phenotype will ultimately need to be determined empirically, although the phenotype of conditional gene knock-outs in adult mice should inform this approach (Regan et al., 2002).

ERK5 seems like an excellent candidate for a PROTAC-based approach. It has a classical ATP-binding kinase catalytic domain, through which it phosphorylates MEF2 transcription factors. However, like most protein kinases it also has other domains that are sites for further regulation by other pathways that are known to play a role in cancer, such as phosphorylation by ERK1/2 and CDK1 (Tusa et al., 2018), or possess non-catalytic functions that are not well targeted by small molecules such as the NLS and the TAD (Lochhead et al., 2020). Most strikingly, the phenotype of selective ERK5 kinase inhibition by AX15836 does not match the phenotype of ERK5 gene knock-out (Lin et al., 2016) and a range of ERK5 kinase inhibitors actually activate non-kinase functions such as the TAD (Lochhead et al., 2020). Given the availability of potent and selective ERK5 binders such as AX15836, compound 46, compound 34b, and BAY-885 a next critical step is to explore their utility as ligands for PROTAC-based, targeted ERK5 protein degradation (Figure 5B). Furthermore, in the context of targeting ERK5 by PROTACs in melanoma or PDAC, careful consideration should be given to seeking an E3 ligase that exhibits selective expression in melanoma or PDAC to mitigate possible on-target toxicity in other tissues. To date the majority of PROTACs in clinical development are for the treatment of cancer, and the majority of these recruit the E3 ligase Cereblon (CRBN) (Bekes et al., 2022), however there are no PROTAC therapies for melanoma or PDAC at this stage and it remains to be determined which E3 ligase(s) will be best.

The clinical success of PROTACs is on the cusp of realisation, with the first PROTAC clinical proof-of-concept data reported in 2020 for the oestrogen receptor in breast cancer, and the androgen receptor in prostate cancer. In total there are 15 PROTACs in clinical development (Bekes et al., 2022).

### Oligonucleotide Therapies

Another strategy to deplete intracellular POIs is to use oligonucleotides (nucleic acid polymers) such as Antisense Oligonucleotides (ASOs) and RNAi. ASOs are small (∼18–30 nucleotides), synthetic, single stranded nucleic acid polymers. They are broadly divided into two categories: RNase H-competent or steric block. RNAi’s have a characteristic double stranded 19 + 2 mer structure which is complementary to the target mRNA and mediates gene silencing *via* the slicing of target mRNA transcripts by the RNA-induced silencing complex (RISC). All modalities lead to the degradation of target mRNA and downregulation of the disease-causing POI, whereas steric block ASO’s can also promote alternative splicing leading to the down regulation of a specific splice variant. They rely on ubiquitously expressed, endogenous enzymes; for ASO’s this is the RNase H enzyme, RNASEH1, and for siRNA Argonaut 2 (ARGO2) [reviewed in (Roberts et al., 2020)].

There are nine approved single-stranded antisense oligonucleotide drugs for a range of diseases including Duchenne muscular dystrophy and cytomegalovirus retinitis, but none currently for cancer (Crooke et al., 2021). However, there are seven antisense oligonucleotide drugs in clinical trials for cancer with the most advanced being Danvatirsen (AZD9150/IONIS-STAT3-2.5_RX_) for treatment of lymphoma and lung cancer, which reduces its target, STAT3, lowering IL-6 in the serum and reducing tumour burden (Hong et al., 2015; Reilley et al., 2018). The full impact of antisense and RNAi technologies in cancer therapy remains to be seen. However, given the success of siRNA and shRNA-mediated ERK5 ablation in pre-clinical models (see above), these approaches could offer the same advantages as PROTACs by downregulating the ERK5 kinase domain, NLS, and TAD or selectively remove ERK5 functional domains by alternative splicing. It remains to be determined which domains of ERK5 have critical therapeutic value in targeting.

## Summary

The last few years has seen a significant advance in our understanding of the role ERK5 in certain malignancies, most notably in melanoma where components of the entire core pathway are amplified or upregulated and where ERK5 activation can drive resistance to BRAF, MEK or ERK1/2 inhibitors. In parallel, advances in ERK5 drug discovery have led to a growing pharmacological “tool kit” to interrogate the role of this pathway. ERK5i that have been reported to date exemplify many of the challenges of developing small molecule protein kinase inhibitors. For example, the off-target activity against BRD4 means XMD8-92, XMD17-109, and XMD17-26 should not be used to evaluate the cellular or *in vivo* role of ERK5 kinase activity. More recent second generation ERK5i that lack BRD4 activity include the dual-ERK5/LRRK2 inhibitors, JWG-045, and JWG-071 and the highly selective ERK5i, AX15836 compound 46, compound 34b, and BAY-885. However, even these more selective ERK5i drive the unanticipated paradoxical activation of the C-terminal ERK5 TAD, highlighting both kinase-dependent, and independent functions of ERK5. The time is right to explore emerging drug modalities that ablate all ERK5 functions; for example, by deploying the most selective ERK5i in PROTAC-based approaches. Comparisons between any given ERK5i and its PROTAC counterpart will prove informative in defining the role of ERK5 in cancer and other disease indications, including inflammation, and will also shed light on the normal biological roles of this enigmatic protein kinase.
